# Will Lipidation of ApoA1 through Interaction with ABCA1 at the Intestinal Level Affect the Protective Functions of HDL?

**DOI:** 10.3390/biology4010017

**Published:** 2015-01-06

**Authors:** Eric J. Niesor

**Affiliations:** F. Hoffmann-La Roche Ltd., Grenzacherstrasse 124, CH-4070 Basel, Switzerland; E-Mail: eric_j.niesor@roche.com; Tel.: +41-79-251-9169

**Keywords:** HDL, cholesterol, lutein, ABCA1, antioxidants, apolipoprotein, HDL metabolism

## Abstract

The relationship between levels of high-density lipoprotein cholesterol (HDL-C) and cardiovascular (CV) risk is well recognized; however, in recent years, large-scale phase III studies with HDL-C-raising or -mimicking agents have failed to demonstrate a clinical benefit on CV outcomes associated with raising HDL-C, casting doubt on the “HDL hypothesis.” This article reviews potential reasons for the observed negative findings with these pharmaceutical compounds, focusing on the paucity of translational models and relevant biomarkers related to HDL metabolism that may have confounded understanding of *in vivo* mechanisms. A unique function of HDL is its ability to interact with the ATP-binding cassette transporter (ABC) A1 via apolipoprotein (Apo) A1. Only recently, studies have shown that this process may be involved in the intestinal uptake of dietary sterols and antioxidants (vitamin E, lutein and zeaxanthin) at the basolateral surface of enterocytes. This parameter should be assessed for HDL-raising drugs in addition to the more documented reverse cholesterol transport (RCT) from peripheral tissues to the liver. Indeed, a single mechanism involving the same interaction between ApoA1 and ABCA1 may encompass two HDL functions previously considered as separate: antioxidant through the intestinal uptake of antioxidants and RCT through cholesterol efflux from loaded cells such as macrophages.

## 1. Introduction

Numerous epidemiological studies have demonstrated an inverse relationship between plasma levels of high-density lipoprotein cholesterol (HDL-C) and cardiovascular (CV) risk [1]. Based on these observations it was postulated that raising plasma HDL-C may be protective against cardiovascular disease (CVD), as an increase in HDL-C may reflect the body’s capacity to return peripheral tissue cholesterol to the liver for elimination [2]. As such, pharmaceutical interventions that raise HDL-C in animal models or in patients have been considered as potential therapeutic approaches to treat CVD [3,4].

In recent years, several HDL-C-raising or HDL-mimicking interventions have failed in outcomes studies [5]; in most cases, patients were optimally treated with statins (*i.e.*, ACS patients) [5,6,7,8,9,10,11]. This series of studies with negative findings of compounds with unrelated mechanisms of action is unlikely to be due to chance and challenges the “HDL hypothesis” [12]. The background treatments that are now standard of care may be a confounding factor since in the case of niacin, some benefit in reduction of non-fatal myocardial infarction was observed; however, this was not reproduced in recent studies in patients optimally treated with statins [13]. It is unlikely that in the near future new compounds that affect only HDL-C levels will reach phase III outcome hallmarks.

Anacetrapib and evacetrapib, two cholesteryl ester transfer protein (CETP) inhibitors that raise HDL-C and are currently in phase III, also dramatically affect low-density lipoprotein cholesterol (LDL-C) levels. The mechanism of the latter effect is as yet unknown and does not involve changes in proprotein convertase subtilisin/kexin type 9 (PCSK9) [14], but may involve a potentially off-target mechanism that interferes with the low-density lipoprotein receptor pathway [15], thus preventing testing of the HDL hypothesis. Dalcetrapib, a CETP modulator [16] which raises HDL-C and apolipoprotein (Apo) A1 (even in patients on statins), remains a main challenge to the HDL hypothesis since no benefit on clinical outcomes was seen in phase III with dalcetrapib [10].

The most common explanations for the failures of HDL-C-raising compounds are side effects (torcetrapib), weak inhibition of CETP (dalcetrapib) and that HDL and its metabolism are extremely complex (*i.e.*, more complex than LDL). This diversity in mode of action in HDL-raising compounds permits further examination of HDL metabolism, shedding new light on its function and implications for the future. In this article, I hypothesize that (1) contrary to popular belief, dalcetrapib is not a “classical” CETP inhibitor since it can also increase CETP activity [16]; (2) the function of HDL can be reduced to a basic interaction between the ATP-binding cassette transporter (ABC) A1 and ApoA1, and (3) that the role of the intestine in generating atheroprotective HDL has been relatively neglected compared with its role in cholesterol efflux capacity from loaded macrophages. Indeed, a unique function of HDL is its ability to interact with ABCA1 via ApoA1. This process may be involved in the intestinal uptake of dietary sterol and antioxidants as well as reverse cholesterol transport from peripheral tissues to the liver.

This article reviews some of the reasons for the failure of HDL-raising intervention, focusing on the lack of translational models for niacin and fibrates that have contributed to the poor understanding of HDL-raising effects *in vivo*, and a paucity of relevant biomarkers related to HDL metabolism from a drug development point of view. Evidence for a more important role for the intestine in generating atheroprotective HDL through ABCA1/ApoA1 interaction is discussed.

## 2. Difficulties in Translational Studies Related to High-Density Lipoprotein (HDL) Metabolism

### 2.1. Absence of Reliable Method to Biochemically Characterize HDL Particles

A lack of standardized or comparable methods to characterize and define HDL has made it difficult to evaluate results from different laboratories [17]. The vast majority of large-scale epidemiology studies and genetic analyses rely on HDL-C plasma levels, which provide only a crude estimate of HDL particle concentration, ApoA levels and HDL function. Proteomic and lipidomic studies have also added to the assumed complexity of HDL. Indeed, more than 100 proteins [18] and thousands of lipids [19,20] are associated with HDL.

### 2.2. Lack of Clinically Relevant Pre-Clinical Animal Models for Drug Development

A paucity of clinically relevant pre-clinical animal models has also confounded attempts to understand the mechanism of action of HDL-raising drugs and to assess new HDL-raising therapies. The two most studied and marketed classes of HDL-raising compound are niacin (and analogs) and fibrates. The HDL-raising activity of niacin treatment was first empirically observed in humans. Indeed, niacin decreases free fatty acids and triglycerides (TG) in almost all species (dogs, mice, rats, guinea pigs and sub-human primates), but only raises HDL-C in primates. Thus, no small animal models have been available to investigate the *in vivo* mechanisms of action of niacin that lead to increased HDL-C. The identification of a putative niacin G-protein coupled membrane receptor, HM74, was surprisingly not linked to HDL increase but mainly to the lipolytic activity of niacin [21] and although many hypotheses have been proposed [22,23], the mechanism and species selectivity of the HDL-raising activity of niacin remains an enigma.

Fibrates, exemplified by fenofibrate, were first described as hypocholesterolemic compounds in rats. This was almost exclusively due to a decrease in HDL-C, the main cholesterol-carrying lipoprotein in rodents. Unexpectedly, however, fenofibrate was observed to increase HDL-C in humans, most likely because human ApoA1 is up-regulated by fibrates, via activation of the peroxisome proliferator-activated receptor alpha (PPAR-alpha), whereas rodent ApoA1 expression is decreased by PPAR activators [24]. Thus, as noted for niacin, fibrates also lack relevant animal models and their mechanism of action *in vivo* has remained poorly understood.

Although CETP is not expressed by rats and mice [25], guinea pigs, hamsters, rabbits and non-human primates do express the CETP gene and transfer activity [26]. The last four are common laboratory animal models providing at least three animal models from a broad range of species [27] in which the *in vivo* effects of CETP inhibitors and modulators, the role of CETP and its interaction with HDL, and the role of different HDL particles in HDL functions [16,28,29] can be investigated. Nevertheless, hamster, rabbit, monkey and human CETP were recently demonstrated to have distinct functional properties [30]. Although the rabbit, hamster and human respond similarly by an increase in HDL-C to CETP inhibitors, such as anacetrapib, the effect of the CETP modulator dalcetrapib differs markedly from anacetrapib in hamsters [29].

### 2.3. Early Indications that High-Density Lipoprotein Cholesterol HDL-C Is Not Always a Reliable Biomarker of Cardiovascular (CV) Risk

There are numerous epidemiological reports demonstrating that in the general population a high level of HDL-C consistently confers protection to cardiovascular diseases, thereby leading to the hypothesis that high levels of HDL-C were related to the mechanism of atheroprotection of HDL and reflect a higher level of cholesterol mobilization from peripheral tissues. Indeed, direct infusion of ApoA1 to human led to an increased mobilization and fecal elimination of cholesterol [31]. Similar results were produced in mice supporting the role of HDL in reverse cholesterol transport. The “HDL-C hypothesis,” led to the concept that raising HDL-C may be necessary to provide CV benefit and more than four decades of research for HDL-C-raising drugs. This approach failed to take into account information available more than 30 years ago with the discovery of subjects with the ApoA1 Milano (ApoA1M) mutation [32]. These subjects, with very low plasma HDL-C levels, do not suffer from any increase in CVD. Thus, very low levels of HDL-C are not necessarily associated with CVD. HDL from ApoA1M does not mature into large HDL, also partly due to a low lecithin-cholesterol acyltransferase activity, and migrates like small poorly lipidated pre-beta HDL [33] with rapid exchange and turnover rate [34]. ApoA1 Paris (ApoA1P) is another ApoA1 mutation producing HDL with kinetic properties similar to ApoA1M [35]. Recently, Gursky *et al.* [36] proposed that the structures of ApoA1M and ApoA1P increase the “exchangeability” of ApoA1 by decreasing its binding to the lipid phase of HDL. This point of view suggests that the exchange rate and “recycling” of ApoA1 may be more important to maintain the HDL protective function than the formation of large HDL.

Very high levels of HDL-C can also be associated with increased CVD. Indeed, studies in mice showed that knocking down the HDL receptor, scavenger receptor class B1 (SRB1), was an efficient way to dramatically increase HDL-C [37]; unfortunately, the raised HDL-C was associated with an increase in atherogenicity. This observation was later confirmed in liver-selective SRB1 knock-out (KO) mice [38], where an increase in atherogenic risk in the presence of high HDL-C was demonstrated. Interestingly, the expression of the CETP gene in the liver of these SRB1 KO mice decreased the atherogenic burden, suggesting that CETP may have a protective role in preventing the formation of very large atherogenic HDL particles, favoring their elimination.

In addition, the relatively high frequency of CETP deficiency in Japan and higher HDL-C levels seen in carriers have not been associated with a clear CV benefit, but instead, major controversy regarding the protective role of high HDL-C levels [39].

Taken together, these observations in ApoA1M subjects, SRB1 KO mice and CETP-deficient individuals suggest that HDL recycling and turnover is more important than plasma HDL-C level *per se*. Recent large-scale investigations [40] support the initial observation made by Khera *et al.* [41], demonstrating that cholesterol efflux capacity of HDL may be a potentially reliable and functional marker of the HDL atheroprotective activity.

### 2.4. Role of Cholesteryl Ester Transfer Protein (CETP) in HDL Recycling and ApoA1 Exchange

CETP has been shown reproducibly to remodel HDL by removing ApoA1 from large HDL particles, in the absence of bidirectional transfer of neutral lipids between HDL and acceptor very low density lipoprotein (VLDL) or LDL [42,43,44]. This activity has been demonstrated using purified HDL and CETP in the complete absence of other lipoproteins. More importantly, Lagrost *et al.* [43] showed that the anti-CETP antibody TP1 was able to prevent CETP-induced HDL remodeling. We have replicated this observation and demonstrated that CETP inhibitors such as torcetrapib and anacetrapib block this process, whereas dalcetrapib [16] and close analogs [45] can activate CETP activity. To date, the only activity of CETP measured in all genetic and clinical studies is the exchange of cholesteryl ester in exchange for TG using an HDL-like donor and VLDL/LDL-like acceptor. This is largely due to the fact that the only commercially available assay to measure CETP activity is the transfer of labeled cholesteryl ester or cholesteryl ester analog to acceptor liposome or VLDL/LDL [46].

## 3. HDL Function: Roles of ABCA1, ABCG1 and SRB1 in Cholesterol Efflux from Macrophages

Until the recent failures of HDL-raising interventions in Phase III outcome studies, most reviews on HDL proposed an array of atheroprotective properties of HDL that should decrease CV risk [47]. With the exception of cholesterol efflux, few of these hypotheses have been tested in the clinical setting. HDL-C efflux of labeled cholesterol from mouse macrophage to HDL from patients has been assessed recently in a number of clinical interventions. Unfortunately, labeled cholesterol can be delivered to plasma components by macrophages *in vitro* though different and more or less selective mechanisms: ABCA1, ABCG1 and SRB1 [48], for which *in vivo* and clinical relevance is not established and which makes the standardization of relevant assays difficult.

### 3.1. Macrophage as Preferred Target Cell for the Antiatherosclerotic/Atheroprotective Role of HDL

#### 3.1.1. ABCA1

The contribution of cellular ABCA1 in the generation of plasma HDL and maintenance of plasma HDL-C levels is well established and well correlated with *in vitro* efflux of tissue cholesterol [49,50]. ABCA1 regulatory elements and variations in gene expression have also been associated with atherosclerosis [49,51,52].

Confusion has surrounded the role and relevance of ABCG1 in mediating cholesterol efflux to large HDL [53]. ABCG1-driven efflux of cholesterol to lipidated HDL has been demonstrated *in vitro* [54]; however, in mice, knocking down ABCG1 does not impact plasma HDL-C as clearly demonstrated for ABCA1. ABCG1 is often visually depicted at the cell surface, mediating efflux directly to large/lipidated HDL particles in a manner similar to ABCA1, but detailed studies revealed that ABCG1 is an intracellular transporter [55]. Furthermore, neither decreased ABCG1 activity nor gain-of-function mutations in humans modify plasma HDL-C as observed for ABCA1 [49,52,53]. Thus, while studies suggest that ABCG1-driven efflux of cholesterol to lipidated HDL occurs *in vitro*, these data strongly suggest that this transporter does not contribute to plasma HDL-C levels and may not have a prominent role in HDL metabolism *in vivo*. Interestingly, while efflux via ABCA1 is selective to ApoA1 or nascent HDL, efflux of cholesterol via ABCG1 *in vitro* is not selective to HDL; LDL, VLDL or liposomes are alternative cholesterol acceptors [56].

More recent studies, which used plasma from patients treated with HDL-raising agents such as torcetrapib [57], niacin and anacetrapib [58], have only contributed to confusion surrounding the choice of assay used to measure efficacy of HDL-raising interventions and resulted in a temptation to place ABCG1 at a functional level equivalent to ABCA1 [59].

Extrapolation and misinterpretation of findings from *in vitro* studies of cholesterol efflux via SRB1 and studies using mature (lipidated) HDL particles have also added to the confusion surrounding the relationship between cholesterol efflux and CV risk. While efflux of labeled cholesterol can be modified by SRB1 expression *in vitro*, knocking down SRB1 *in vivo* (in mice) not only does not decrease HDL-C (as would have been expected if SRB1 efflux were contributing to plasma HDL-C levels), but dramatically increases HDL-C and atherosclerosis [37]. Interestingly, humans with moderate SRB1 deficiency who carry a P297S mutation show increased plasma HDL-C levels (70.4 mg/dL *vs.* 53.4 mg/dL in non-carriers; *p* < 0.001), but a reduced capacity for cholesterol efflux from macrophages [60]. SRB1 may be more involved in uptake of cholesterol (for example by adrenal cells) than in cholesterol efflux. Indeed, cholesterol uptake from HDL by primary murine hepatocytes expressing mutant SRB1 is reduced to half that seen in hepatocytes expressing wild-type SRB1. A decrease in adrenal steroidogenesis further supports this hypothesis [61].

#### 3.1.2. Macrophage Cholesterol Efflux as a Potential Marker of HDL Function and CV Risk

Khera *et al.* demonstrated that *in vitro* cholesterol efflux might be a better biomarker of CV risk than HDL-C [41]. The cholesterol-labeled macrophage efflux assay has been investigated in numerous clinical studies assessing HDL-raising or -mimicking agents. Importantly, pioglitazone [41], niacin and anacetrapib [58], torcetrapib [62], and CER-001 [63] have been shown to produce HDL with maintained or improved efflux capacity. More recently, we showed that the HDL raised by dalcetrapib in patients during and in the months following an acute coronary syndrome (ACS) displayed an increased efflux capacity in spite of the inflammatory milieu characteristic of ACS [64]. Thus far, for almost all HDL-raising or -mimicking interventions, positive efflux data were obtained *in vitro*.

It should be noted that efflux to ApoB-depleted serum from patients, measured *in vitro*, does not take into account *in vivo* tissue ABCA1 expression and activity. For example, decreased ABCA1 activity *in vivo* may lead to an increase in non/poorly lipidated pre-beta-1 HDL which, when exposed to mouse macrophages with normal, cAMP or liver X receptor (LXR)-activated ABCA1 expression, will display a higher efflux capacity than ApoB-depleted plasma from subjects with normal ABCA1 function. This may explain the recent unexpected observation made by Li *et al.* [65] of a higher efflux capacity associated with increased CV risk and the increased capacity of HDL to efflux cholesterol via ABCA1 in hypertriglyceridemia [66] and type 2 diabetes [67], which are both risk factors for CVD.

## 4. HDL Function at the Intestinal Level

### 4.1. Uptake of Cholesterol

ABCA1 deficiency is associated with almost complete absence of plasma HDL-C in all species examined: human [68], mice [51] and chicken [69]. Tissue-selective ABCA1 KO mice have decreased plasma HDL-C in the particular tissue, with no compensation by other tissues. For example, liver and intestinal ABCA1 KO mice have markedly reduced levels of HDL-C (60% and 30%, respectively) [70]. However, plasma HDL-C is not significantly modified in adipose tissue [71] and macrophage ABCA1 KO [72]. As such, ABCA1 expression/activity in individual tissues contributes in a complex manner to plasma HDL-C levels, with tissue cholesterol stores a confounding factor. Recently, Iqbal *et al.* [73] demonstrated that cholesterol is absorbed by two independent pathways: (1) involving microsomal triglyceride transfer, acetyl-coenzyme A acetyltransferase 2 and secreted as chylomicrons; and (2) involving ABCA1/ApoA1 interaction and secreted as HDL particles. It should be noted that these data were obtained in mice and that the contribution of the intestine to the absorption cholesterol through lipidation of HDL in human is not known.

### 4.2. Uptake of Phytosterols

Since measurable amounts of cholesterol are absorbed at the intestinal level through the ABCA1/ApoA1 efflux system [74], we hypothesized that the absorption of phytosterol (which cannot be synthetized by animals) via the HDL pathway could be used as a marker of intestinal ABCA1/ApoA1 activity.

We have previously shown that plant sterols, which offer the advantage of being strictly of dietary origin, are absorbed at the intestinal level via a HDL pathway [28], very likely during and with a similar process as ApoA1 lipidation by cholesterol and related to pre-beta1 HDL levels [75]. The original observation [28] was made both in hamsters and healthy human volunteers treated with the CETP modulator dalcetrapib, which affects HDL metabolism in both species. Similar observations of higher plasma phytosterol levels have been made in patients with high HDL-C levels (matched for similar LDL-C levels) [76] and patients with high HDL-C levels due to exercise [77]. Conversely, however, in the PROCAM (Prospective Cardiovascular Münster) study, patients with low HDL-C levels displayed decreased plasma phytosterol levels with a direct correlation between parameters [78].

**Figure 1:**
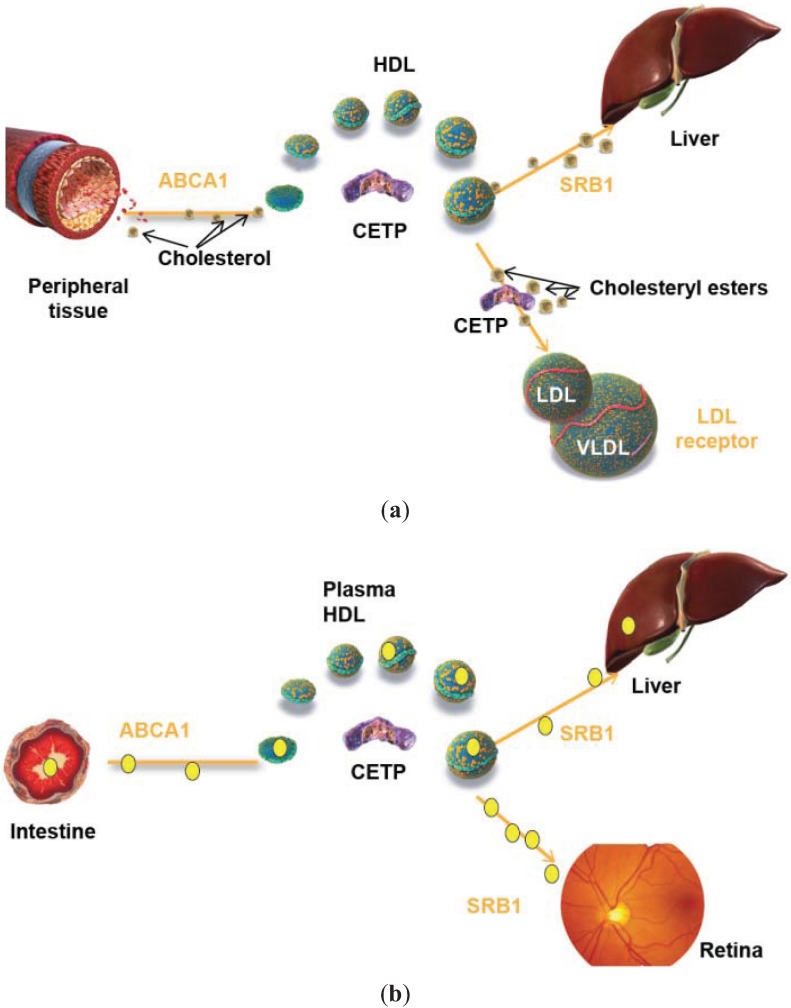
(**a**) HDL-mediated efflux of cholesterol from macrophages of atherosclerotic arteries and (**b**) HDL-mediated uptake of xanthophylls (yellow dots) by enterocytes. ABCA1 = ATP-binding cassette transporter A1; CETP = cholesteryl ester transfer protein; HDL = high-density lipoprotein; LDL = low-density lipoprotein; SRB1 = scavenger receptor class B1; VLDL = very low-density lipoprotein.

Phytosterol absorbed via the HDL pathway requires functional ABCA1 and ApoA1; an HDL-dependent increase in plasma phytosterol was not observed in dalcetrapib-treated patients with mutations in ApoA1 and/or ABCA1 [79]. Dalcetrapib increased HDL-C and ApoA1 to a similar extent in patients with low HDL-C resulting from mutations (*i.e.*, familial hypoalphalipoproteinemia; FHA) and patients with low HDL-C not arising from mutations (*i.e.*, familial combined hyperlipidemia); however, patients with FHA did not have an increase in plasma campesterol. It should be noted that the increase in plasma phytosterol triggered by dalcetrapib treatment or measured in high HDL subjects is in a similar range to the increase measured in statin-treated patients [80] and different from those measured in ABCG5/G8 mutation and leading to atherogenic phytosterolemia [81]. Based on these results, we proposed (Figure 1) that phytosterols not returned to the intestinal lumen via ABCG5/G8 activity are absorbed by the chylomicron pathway with trace amounts absorbed via an HDL pathway and very likely eliminated efficiently by liver as proposed by Robins and Fasulo [82].

### 4.3. Uptake of Xanthophyll

HDL has been recognized to have antioxidant properties and to protect LDL from oxidation *in vivo* [83] and *in vitro* [84]. This potentially atheroprotective activity has been attributed to enzymes bound to HDL, such as paraoxonase 1 (PON1) and matrix metallopeptidase 2 (MMP2) or antioxidants such as vitamin E, lutein and zeaxanthin. Decreased PON1 activity has been associated with the antioxidant and atheroprotective activity of HDL [85] and decreased in low-HDL subjects [86]; however, the effect of HDL-C-raising interventions on this process has been controversial for fenofibrate. The effect of fenofibrate therapy on paraoxonase1 status in patients with low HDL-C levels [87] induces HDL-associated PAF-AH but attenuates enzyme activity associated with apoB-containing lipoproteins [88]. It has been recently demonstrated that oxidation by myeloperoxidase [89], or other selective modifications of ApoA1, destroys some of the key protective properties of ApoA1 (cholesterol efflux) [90] and increases the risk of CVD [91]. This raises the question of the adequate amount of antioxidant enzymes: PON1 and MMP2, vitamin E and xanthophylls in HDL to be functionally atheroprotective [92].

Hirowatari *et al.* [93] showed that the relative amount of the vitamin E isomers alpha plus gamma tocopherol in HDL is 5.82 nmol/mol cholesterol, whereas it is only 3.55 in LDL. Similarly, lutein and zeaxanthin are preferentially transported by HDL: the ratio of lutein and zeaxanthin in HDL:LDL is 2.49 and 1.95, respectively. For alpha and beta-carotene these ratios are 0.45 and 0.31, respectively [94]. As expected from their relative lipophilicity, the bulk of tocopherol and carotene are transported by LDL (45% and 76%, respectively), whereas the oxygenated carotenoids (lutein, zeaxanthin, canthaxanthin and beta-cryptoxanthin) are equally distributed between LDL and HDL [95]. Thus, HDL preferentially carries a number of lipophilic antioxidants (vitamin E, lutein and zeaxanthin) that very likely contribute to its antioxidative properties. This has been demonstrated *in vitro* by the capacity of HDL enriched with these antioxidants to protect LDL from oxidation [96]. Since vitamin E, lutein and zeaxanthin are exclusively obtained from the diet and transported by plasma lipoproteins, pathways involved in their intestinal absorption are of major importance.

Until recently, these antioxidants were thought to be absorbed exclusively via a chylomicron-mediated pathway [97] and were assumed to be redistributed among lipoproteins during secretion of VLDL by the liver, or via an exchange proteins such as CETP [98] or phospholipid transfer protein (PLTP) [99], as is the case for vitamin E. Xanthophylls may also be exchanged between lipoproteins by CETP [100]; however, *in vivo* evidence for exchange through such a mechanism is still lacking.

Using Caco-2 cells, Nicod *et al.* [101] demonstrated that vitamin E is also absorbed and directly delivered to ApoA1 via ABCA1, not to mature HDL. *In vivo* studies using ABCA1 KO mice demonstrated that plasma gamma-tocopherol levels were four-fold lower in ABCA1 KO mice than in wild-type mice, whereas retinyl ester plasma levels did not differ between animal strains [97]. The mechanism appeared to be enantioselective, since alpha-tocopherol and gamma-tocopherol were preferentially absorbed compared with delta-tocopherol [101]. Thus, although the amount of vitamin E delivered directly to plasma HDL may be less compared to the chylomicron pathway; it may be more selective and may allow delivery to tissues that do not express the ApoB receptor, but express instead the HDL receptor SRB1.

We followed a similar reasoning for the absorption of the important antioxidative xanthophylls, lutein and zeaxanthin, knowing that it has been shown previously that Wisconsin HypoAlpha Mutant (WHAM) chickens with ABCA1 loss of function mutations have negligible plasma and retinal lutein, but still maintain liver and egg yolk lutein levels [102]. Using hamsters fed a lutein and zeaxanthin supplement, we demonstrated that after treatment with dalcetrapib, which increases the remodeling of HDL to produce free ApoA1, lutein and zeaxanthin uptake were increased [29]. Although the CETP inhibitor anacetrapib markedly increased HDL-C in the same experiment, there was no change in plasma and liver lutein levels. This study demonstrated that HDL-C levels can be disconnected from the level of antioxidants transported in the plasma of these animals which contain mainly HDL particles as plasma lipoproteins.

**Figure 2:**
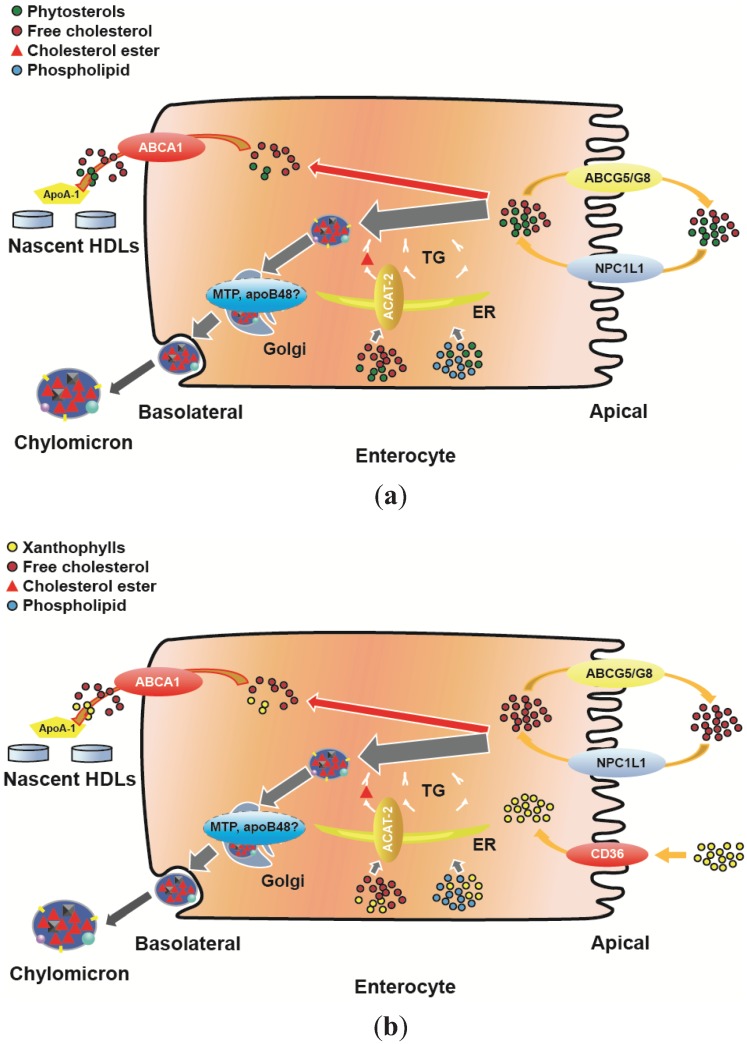
(**a**) Contribution of intestinal cholesterol, phytosterol and ACAT-2 to ApoA1 lipidation and (**b**) Contribution of xanthophylls and ACAT-2 to ApoA1 lipidation. ABCA1 = ATP-binding cassette transporter A1; ABCG5/G8 = ATP-binding cassette transporter G5/G8; ACAT-2 = acyl-coenzyme A: cholesterol acyltransferase 2; apoA1 = apolipoprotein A1; apoB48 = apolipoprotein B48; CD36 = cluster of differentiation 36; ER endoplasmic reticulum; FABP = fatty-acid binding protein; HDL = high-density lipoprotein; MTP = microsomal triglyceride transfer protein; NPC1L1 = Niemann-Pick C1-Like 1; SRB1 = scavenger receptor class B1; TG = triglycerides.

Interestingly, in the hamster model, treatment with the LXR agonist T0901317 dramatically increased plasma and liver lutein and intestinal ABCA1 messenger RNA (mRNA) [29]. Intestinal ABCA1 mRNA was well correlated with plasma lutein levels, supporting the hypothesis that, in addition to the chylomicron pathway, a significant portion of intestinal xanthophylls are absorbed via an ABCA1/ApoA1 pathway and may be preferentially delivered to some tissues, such as the retina. Simvastatin treatment, which modestly decreased intestinal ABCA1 mRNA, dramatically decreased plasma and liver lutein and zeaxanthin without affecting either HDL-C or LDL-C levels. This suggested that delivery of xanthophyll via ABCA1 is very sensitive to a small decrease in expression and raised the question of the effect of statin treatment on HDL antioxidant levels—to our knowledge, this has never been reported. As described for cholesterol (Figure 2a), we propose that xanthophylls are absorbed at the intestinal level and secreted at the basolateral surface of enterocytes via a chylomicron-mediated as well as an HDL-mediated pathway (Figure 2b) which can be up- or down-regulated by drug interventions, such as T0901317, dalcetrapib or statins.

From the observations made in the ABCA1 mutant WHAM chicken, in which liver and egg lutein/zeaxanthin levels are normal, it would appear that liver lutein is not delivered to HDL but very likely only secreted with VLDL. Since chicken do express CETP and PLTP [103], the absence of xanthophylls in their plasma cannot be explained by lack of exchange protein. It remains to be determined if liver can secrete xanthophylls via an ABCA1/ApoA1 mechanism as described by Shiriri *et al.* for vitamin E [104].

## 5. HDL Functionality at the Intestinal Level

Investigations in mice in which intestinal ABCA1 was selectively knocked down demonstrated that this pathway contributes to approximately 30% of plasma HDL levels, with no compensation from other tissues [105]. The importance of this pathway has been reviewed recently [74]. Based on recent results demonstrating that traces of plant sterols are also taken up by this pathway and that, more importantly, lipophilic antioxidant vitamin E [97], and lutein and zeaxanthin [29] of dietary origin are directly delivered to ApoA1 via ABCA1, we propose that their delivery to ApoA1 by the intestine contributes to the formation of nascent HDL. Moreover, during this process, sterols and antioxidants are recovered from the intestine and potentially delivered to peripheral tissues, bypassing the liver. Pathological situations and/or drug interventions may affect the capacity of the intestine to deliver antioxidants to HDL and thus contribute to “dysfunctional” HDL (*i.e.*, HDL that cannot protect endothelial cells or LDL from oxidation). It is interesting to note that HDL from patients with ACS have been consistently shown to be “dysfunctional” and that in these studies the vast majority of patients are receiving optimal doses of statin [106]. Studies by Navab *et al.* [107] provide evidence that HDL isolated from patients suffering from different pathologies lack the functions of HDL from healthy subjects. In the Los Angeles Heart Study [96], plasma HDL lutein and its relationship with CVD was not specifically investigated. Vitamin E is a weaker antioxidant than lutein or zeaxanthin [96], and may have a pro-oxidant effect on HDL [108], which could explain the failure of clinical studies to demonstrate a benefit of vitamin E supplementation in protection from CVD. The effect of the marketed HDL-raising drugs fenofibrate and niacin on plasma xanthophylls levels had not been measured until recently [75]. In a crossover study in 66 dyslipidemic subjects treated for 6 weeks with fenofibrate (160 mg/day) or extended-release (ER) niacin (0.5 g/day for 3 weeks then 1 g/day), both treatments increased HDL-C (16%) and ApoA1 (7%), but lutein and zeaxanthin levels were unaffected by fenofibrate and increased by ER-niacin (each ~30%) without any correlation to lipoprotein or Apo levels. Although fenofibrate and ER-niacin similarly increased plasma HDL-C and ApoA1, effects on plasma xanthophylls differed markedly, suggesting differences in intestinal lipidation of HDL. Plasma xanthophylls levels in niacin-treated patients simultaneously receiving statin treatment could be informative. A potential atheroprotective effect of lutein and zeaxanthin in large-scale clinical studies remains to be demonstrated.

## 6. A Key Role of HDL of Intestinal Origin in HDL Protection?

Recent findings support the proposal by Attie *et al.* in 2007 that ABCA1 may indeed be at the center of the relationship between cholesterol, HDL and atherosclerosis [109] and go further to suggest that a single mechanism involving the same interaction between ApoA1 and ABCA1 may encompass two HDL functions previously considered as separate: (1) efflux of cholesterol from macrophages in atherosclerotic arteries (Figure 1a); and (2) uptake of xanthophylls at the basolateral surface of enterocytes (Figure 1b).

Compared to the chylomicron pathway, the HDL pathway is clearly quantitatively less important than the route involving chylomicron but may be qualitatively more selective in its absorption process (*i.e.*, enantioselectivity for tocopherol alpha and gamma *versus* delta) and more importantly in its delivery to specific tissues as observed in SRB1 KO mice [110]. Whether similar enantioselectivity and delivery to tissues applies to xanthophylls is worth investigating, since the plasma:tissue ratio of lutein and zeaxanthin differs from that in the diet. Such investigations may have potential impact in the prevention of age-related macular degeneration [111] and Alzheimer’s disease [112].

**Figure 3:**
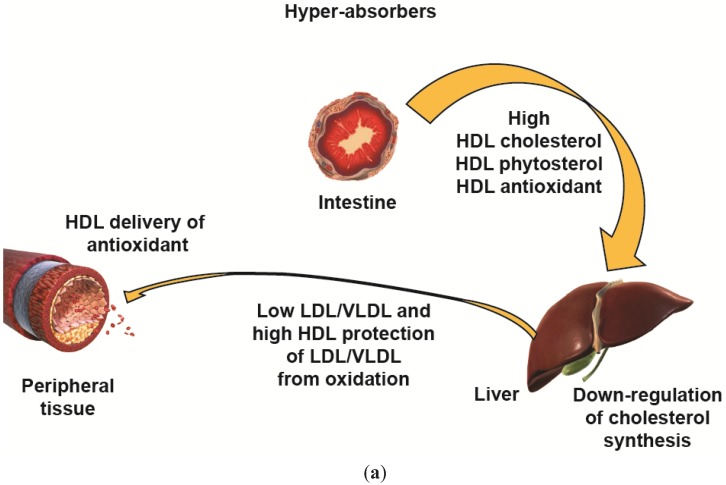

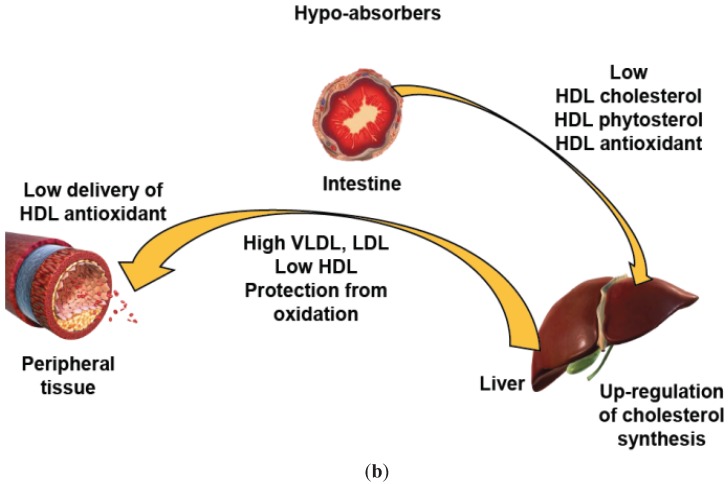
HDL-mediated cholesterol and antioxidant uptake and transport from intestine to peripheral tissues in (**a**) patients with high HDL-C: hyper-absorbers and (**b**) patients with low HDL-C: hypo-absorbers. DL = high-density lipoprotein; LDL = low-density lipoprotein; VLDL = very low-density lipoprotein.

Patients with high HDL-C may be protected from CVD because they are “hyper-absorbers” (*i.e.*, cholesterol is efficiently collected from the intestine and preferentially delivered to the liver for bile acid synthesis). This will down-regulate cholesterol synthesis. The same process will load HDL with antioxidants such as vitamin E, lutein and zeaxanthin, which will prevent LDL and tissue lipid oxidation (Figure 3a). On the contrary, patients with low HDL-C will collect intestinal cholesterol and antioxidants inefficiently; this will trigger cholesterol synthesis mainly at the liver level and initiate the cycle of increased VLDL, LDL-C, *etc.* (Figure 3b). Thus, HDL-C level may be a surrogate marker of intestinal ApoA1 synthesis and lipidation, and HDL lutein and zeaxanthin levels may be more relevant to the atheroprotective and antioxidative properties of HDL. Thus far, this parameter has not been thoroughly investigated in any clinical study involving HDL-raising or -mimicking interventions in patients optimally treated with statins and could be extremely informative.

## 7. Summary

In summary, a single mechanism involving a unique ability to interact with ABCA1 via ApoA1 may underlie both the antioxidative function of HDL via intestinal uptake of xanthophylls at the basolateral surface of enterocytes, and its role in the efflux of cholesterol from loaded cells such as macrophages in atherosclerotic arteries. Further investigation of this hypothesis may provide a better understanding of HDL functionality and provide a positive perspective towards addressing CVD and other degenerative diseases through reinforcing/improving HDL properties.
